# The G Protein Biased Small Molecule Apelin Agonist CMF-019 is Disease Modifying in Endothelial Cell Apoptosis *In Vitro* and Induces Vasodilatation Without Desensitisation *In Vivo*

**DOI:** 10.3389/fphar.2020.588669

**Published:** 2021-01-21

**Authors:** Cai Read, Duuamene Nyimanu, Peiran Yang, Rhoda E. Kuc, Thomas L. Williams, Christopher M. Fitzpatrick, Richard Foster, Robert C. Glen, Janet J. Maguire, Anthony P. Davenport

**Affiliations:** ^1^ Department of Medicine, Experimental Medicine and Immunotherapeutics, Addenbrooke’s Hospital, University of Cambridge, Cambridge, United Kingdom; ^2^ School of Chemistry and Astbury Centre for Structural Biology, University of Leeds, Leeds, United Kingdom; ^3^ Department of Chemistry, Centre for Molecular Informatics, University of Cambridge, Cambridge, United Kingdom; ^4^ Division of Systems Medicine, Department of Metabolism Digestion and Reproduction, Imperial College London, London, United Kingdom

**Keywords:** apelin, bias, cardiovascular, *in vivo*, apoptosis, pulmonary artery endothelial cell, G protein coupled receptor

## Abstract

Signaling through the apelin receptor is beneficial for a number of diseases including pulmonary arterial hypertension. The endogenous small peptides, apelin and elabela/toddler, are downregulated in pulmonary arterial hypertension but are not suitable for exogenous administration owing to a lack of bioavailability, proteolytic instability and susceptibility to renal clearance. CMF-019, a small molecule apelin agonist that displays strong bias towards G protein signaling over β-arrestin (∼400 fold), may be more suitable. This study demonstrates that in addition to being a positive inotrope, CMF-019 caused dose-dependent vasodilatation *in vivo* (50 nmol 4.16 ± 1.18 mmHg, **p < 0.01; 500 nmol 6.62 ± 1.85 mmHg, **p < 0.01), without receptor desensitization. Furthermore, CMF-019 rescues human pulmonary artery endothelial cells from apoptosis induced by tumor necrosis factor α and cycloheximide (5.66 ± 0.97%, **p < 0.01) by approximately 50% of that observable with rhVEGF (11.59 ± 1.85%, **p < 0.01), suggesting it has disease-modifying potential *in vitro*. CMF-019 displays remarkable bias at the apelin receptor for a small molecule and importantly recapitulates all aspects of the cardiovascular responses to the endogenous ligand, [Pyr^1^]apelin-13, *in vivo.* Additionally, it is able to protect human pulmonary artery endothelial cells from apoptosis, suggesting that the beneficial effects observed with apelin agonists extend beyond hemodynamic alleviation and address disease etiology itself. These findings support CMF-019 as a G protein biased small molecule apelin agonist *in vitro* and *in vivo* that could form the basis for the design of novel therapeutic agents in chronic diseases, such as, pulmonary arterial hypertension.

## Introduction

The apelin receptor, a class A G protein coupled receptor (O’Dowd et al., 1993) has two endogenous peptide ligands, apelin (Tatemoto et al., 1998) and elabela/toddler (ELA) (Chng et al., 2013; Pauli et al., 2014). The apelin system is a therapeutic target in diseases (Read et al., 2019), such as diabetes (Castan-Laurell et al., 2012), fibrosis (Huang et al., 2016), heart failure (Berry et al., 2004; Jia et al., 2006; Atluri et al., 2007; Koguchi et al., 2012; Pang et al., 2014) and pulmonary arterial hypertension (PAH) (Falcão-Pires et al., 2009; Yang et al., 2015). In PAH, apelin (Goetze et al., 2006; Alastalo et al., 2011; Kim et al., 2013) and ELA (Yang et al., 2017) are downregulated but receptor expression is maintained (Andersen et al., 2009; Falcão-Pires et al., 2009) therefore replacing the missing ligands could be a therapeutic strategy. In a model of PAH [Pyr^1^]apelin-13 (Falcão-Pires et al., 2009; Maguire et al., 2009) and ELA-32 (Yang et al., 2017) prevented disease onset, however, both lack oral bioavailability and are susceptible to proteolytic cleavage and renal excretion. Furthermore, repeated agonist stimulation leads to β-arrestin recruitment and receptor internalization, potentially blunting therapeutic efficacy.

A preferable strategy is to identify G protein biased apelin ligands (Brame et al., 2015). We have identified a small molecule, CMF-019 (Hachtel, et al., 2014), that is a positive inotrope *in vivo* and possesses strong G protein bias. CMF-019 has nanomolar affinity for the apelin receptor in both human and rat heart and whereas this compound inhibits Gα_i_ mediated cAMP accumulation with sub-nanomolar potency comparable to [Pyr^1^]apelin-13 it is over two orders of magnitude less efficient in recruiting β-arrestin or inducing apelin receptor internalization compared to the endogenous agonist (Read et al., 2016). In this study we have investigated whether CMF-019 alters apoptosis in human pulmonary arterial endothelial cells (PAECs), a driver of early disease phase (Wilson et al., 1992; Rabinovitch 2012). We aimed to confirm that a G protein biased compound produces vasodilatation *in vivo*, (the main mechanism of action for most current PAH therapies) without desensitization, as recent studies have suggested that apelin-mediated vasodilatation may occur via β-arrestin signaling (El Messari et al., 2004; Iturrioz et al., 2010; Ceraudo et al., 2014).

## Materials and Methods

### Materials

Chemicals were obtained from Sigma Aldrich Co. Ltd. (Poole, United Kingdom) unless otherwise stated [Pyr^1^]apelin-13 (purity >98%) was from Severn Biotech (Kidderminster, United Kingdom). CMF-019 was synthesized as a potassium salt (Read et al., 2016), initially in the School of Chemistry, University of Leeds (purity > 95%) and later by Tocris (purity 99.3%) (Bristol, United Kingdom). All animal care and rodent experiments complied with the Home Office (United Kingdom) guidelines under the Animals (Scientific Procedures) Act 1986 Amendment Regulations (SI 2012/3,039) and were approved by the local ethics committee (University of Cambridge Animal Welfare and Ethical Review Body).

### Rescue of Human Pulmonary Artery Endothelial Cell Apoptosis

The effects of CMF-019 on endothelial cell apoptosis were tested and compared with recombinant human vascular endothelial growth factor (rhVEGF; R&D Systems, Minneapolis, MN, United States) using human PAECs (Lonza; Cambridge, United Kingdom; *n* = 5 donors: 1 (lot#0000479486), 2 (lot#4F3041), 3 (lot#4F3034), 4 (lot#000657513) and 5 (lot#0000662151), passages 4–6) as previously described (Long et al., 2015) following protocol optimization. Briefly, PAECs were seeded in six-well tissue culture plates at 200,000 cells/well in endothelial growth medium-2 (EGM-2; Lonza, PromoCell; United Kingdom) with 10% fetal bovine serum (FBS, Gibco™, NY, United States) and allowed to attach. On the next day, wells were washed with PBS and the media changed to either endothelial basal medium 2 (EBM-2; Lonza, PromoCell) with 2% FBS or 10% FBS controls. CMF-019 (1–10 µM) or rhVEGF (10 ng/ml) were added to the wells and incubated for 18 h. Apoptosis was induced by incubating the cells with tumor necrosis factor α (TNFα; R&D Systems, 1.5 ng/ml) and cycloheximide (CHX; 20 μg/ml) for 5 h in the experimental wells. Control wells did not receive TNFα/CHX treatment. Cells were then washed in PBS, trypsinized (Lonza), transferred into 1x binding buffer for the apoptosis assay (Thermoscientific; Waltham, MA, United States) and stained with anti-annexin FITC-conjugated antibody (1:2 stock dilution) and propidium iodide (PI, 20 μg/ml) for 15 mins at room temperature. Cells were filtered through 50 µM filters (Sysmex/Partec; Görlitz, Germany) and kept on ice before flow cytometry (Canto II, BD Biosciences; San Jose, CA, United States). For each condition 10,000 events were recorded. Data analysis was performed on FlowJo v10 (FlowJo LLC; Ashland, OR, United States). Annexin^+^/PI^+^ cells were classified as “dead,” Annexin^+^/PI^−^ cells “apoptotic” and Annexin^−^/PI^−^ as “healthy.” Gates were adjusted such that approximately equal numbers of “healthy” and “apoptotic” cells occurred in the TNFα/CHX treated group as this provided a large window for either further induction or rescue of apoptosis. The raw percentage of cells in each gate were used in the data analysis and some variability in the basal amount of apoptotic induction between replicates was observed. A matched ANOVA was utilized to remove this variability and compare data trends.

### *In vivo* Catheterization to Assess Cardiovascular Responses to CMF-019 in Normotensive Male Sprague-Dawley Rats

**FIGURE 1:**
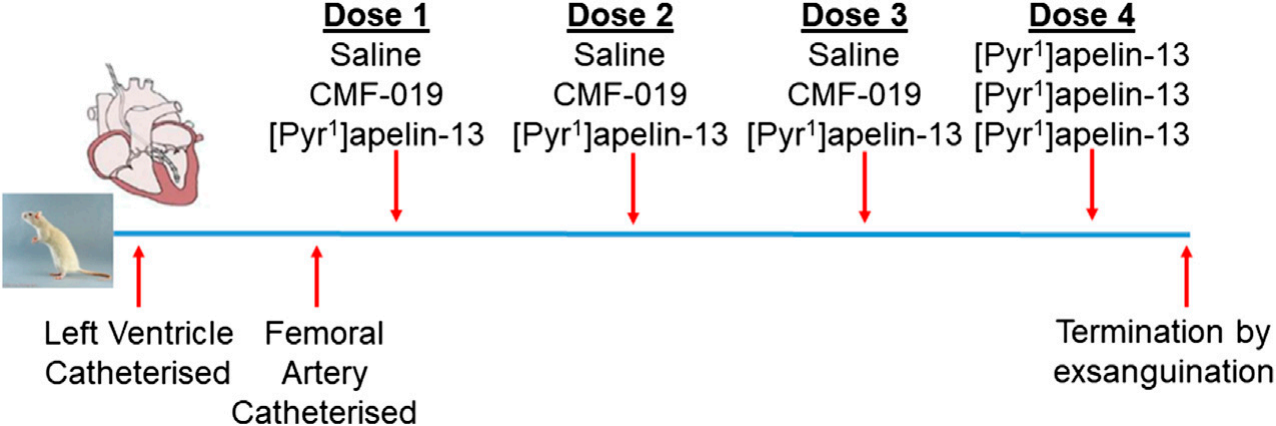
Dosing schedule for the second *in vivo* study. The left ventricle and femoral artery were catheterized in normotensive male Sprague-Dawley rats. Subsequently, the animals were randomly chosen to receive either three doses of saline, CMF-019 or [Pyr^1^]apelin-13, before a fourth dose of 50 nmol [Pyr^1^]apelin-13 was administered regardless of the previous doses administered. Doses were administered at 10 mins intervals or when a stable baseline was reached after which the animal was terminated by exsanguination under high flow isoflurane.

Normotensive male Sprague-Dawley rats (271 ± 3 g, *n* = 17) underwent left ventricular and femoral artery catheterization to assess cardiac and vascular changes upon bolus CMF-019 administration. Left ventricular catheterization was performed as previously described (Pacher et al., 2008; Read et al., 2016; Yang et al., 2017) and the femoral catheterization protocol was developed as an extension to this protocol. In brief, rats were anaesthetized with gaseous isoflurane (3–2.5% for initial induction, maintenance and surgery, and 1.5% for hemodynamics measurement; 1.5 L/min oxygen). The right external jugular vein was exposed, cannulated and flushed with heparin solution (2%) made up in saline (0.9% saline, pH5, Macopharma; Tourcoing, FR). The right common carotid artery was then located and a catheter (SPR-869, Millar Inc.; Houston, TX, United States) inserted and advanced to the left ventricle. Once a stable pressure-volume loop could be observed, the femoral artery was exposed and a second identical catheter inserted to record arterial pressure responses simultaneously to the ventricular responses. Three successive doses of either CMF-019 (50–5000 nmol, 0.5 ml, 0.9% saline, pH9), [Pyr^1^]apelin-13 (10–150 nmol, 0.5 ml, 0.9% saline, pH5) or saline controls were then administered intravenously via the jugular vein catheter, followed by a saline flush (0.9%, 0.1 ml, pH5) at a minimum of 10 mins intervals or when a stable baseline was reached before the next injection. All animals received a 50 nmol dose of [Pyr^1^]apelin-13 as a fourth dose after they had received their first three doses whether they be saline, CMF-019 or [Pyr^1^]apelin-13. The dosing schedule is summarized in Figure 1. Data were acquired using the MPVS Ultra system (ADIstruments; Dunedin, NZ) and analyzed using LabChart 8 (ADIstruments). Values for the maximal change in arterial pressure, left ventricular systolic pressure (LVSP), stroke volume, cardiac output, heart rate, contractility (dP/dt_MAX_) and lusitropy (dP/dt_MIN_) from baseline were calculated from the raw data and compared. Following completion of the measurements the animal was euthanized by exsanguination under high flow isoflurane (5%).

### Statistical Analysis

All data are expressed as mean ± SEM values and statistical analyses were performed with GraphPad Prism 6 (La Jolla, CA, United States) unless otherwise stated. For rescue of human PAEC apoptosis, experiments were performed at least in triplicate. Donors four and five were excluded from the analysis as they showed a particularly small window of apoptotic cell induction using TNF-α and CHX (2.80 ± 1.60%), this was not significantly different to the EBM-2 2% FBS control (Matched ANOVA). Of the remaining donors, technical replicates were excluded if the apoptotic induction was less than 5%. The average induction of apoptosis of the analyzed experiments was 19.53 ± 1.76%. Data were normally distributed using a D’Agostino-Pearson omnibus K^2^ test and trends were compared using a matched ANOVA to account for variability observed in the basal amount of apoptotic induction observed between replicates. For the acute *in vivo* studies, normality has been confirmed by analyzing data collected over a number of experiments using the D’Agostino-Pearson omnibus K^2^ test. Consequently, cardiovascular parameters measured in saline were compared to CMF-019 and [Pyr^1^]apelin-13 treated animals using a two-tailed Student’s t-test. Statistical significance was taken as 5%.

## Results

### CMF-019 Rescued Human PAEC Apoptosis Induced by TNFα and CHX

**FIGURE 2:**
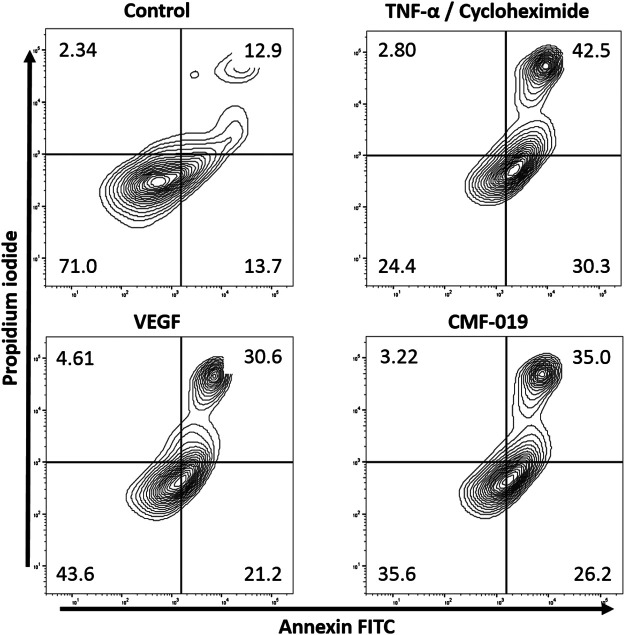
Representative contour plot flow data of human PAECs induced to apoptosis with TNFα/CHX and rescued with either rhVEGF or CMF-019 at 1 µM. Cells were incubated in EGM-2 with 10% FBS (control); EGM-2 with 2% FBS and TNFα/CHX (TNFα/CHX) treatment for 5hrs to induce apoptosis before rhVEGF (10 ng/ml) or CMF-019 (1–10 μM) was added for a further 18 h. PI staining is displayed on the *x*-axis and annexin-V FITC staining on the *y*-axis. The percentage of cells in each quadrant are shown in the corners of the quadrant.

**FIGURE 3:**
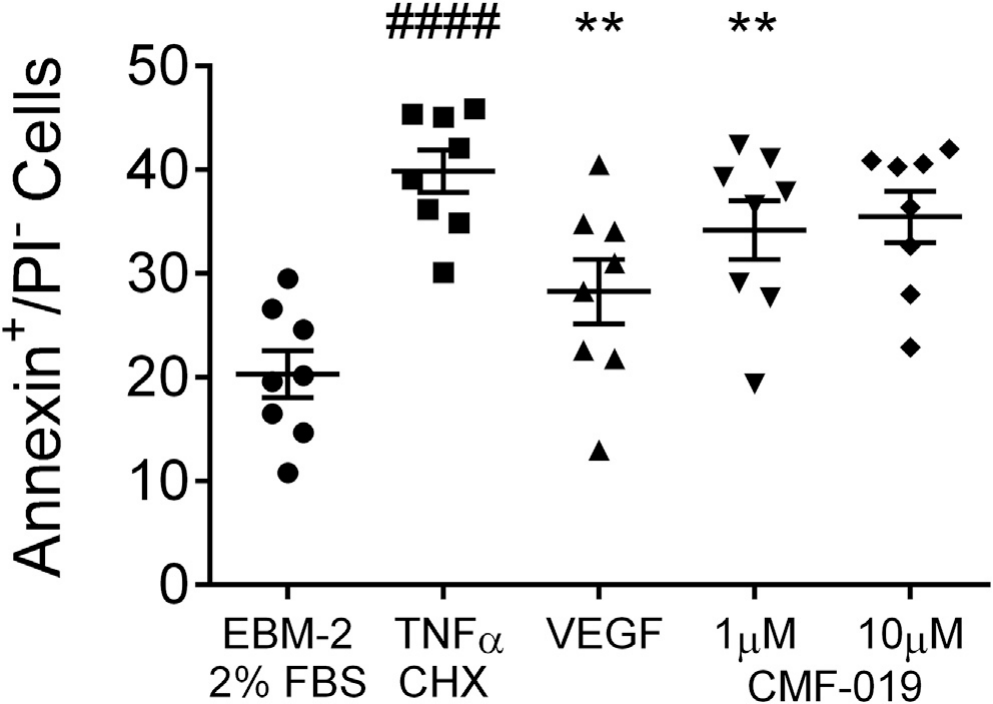
Dot plot of the percentage of annexin^+^/PI^−^ human PAECs in each experimental condition with mean ± SEM data superimposed. Cells were incubated in EGM-2 with 2% FBS alone (control) or EGM-2 with 2% FBS and treated with TNFα/CHX (TNFα/CHX) for 5hrs to induce apoptosis before rhVEGF (10 ng/ml), or CMF-019 (1–10 μM) was added to TNFα/CHX treated cells for a further18hrs. TNFα/CHX significantly increased the percentage of annexin^+^/PI^−^ human PAECs and this could be rescued by 10 ng/ml rhVEGF and CMF-019 at 1 µM. Matched ANOVA comparing each condition to TNFα/CHX **p < 0.01. #### indicates *p* < 0.0001 compared to control (EBM-2 2% FBS).

The ability of CMF-019 to prevent TNFα/CHX induced apoptosis in human PAECs was tested at 1 and 10 µM (Figures 2, 3). TNFα/CHX significantly increased the percentage of annexin^+^/PI^−^ cells (19.54 ± 1.76%, ^####^ *p* < 0.0001) compared to the EBM-2 2% FBS control and rhVEGF was able to partially rescue this (11.59 ± 1.85%, **p < 0.01), as was CMF-019 at 1 µM (5.66 ± 0.97%, **p < 0.01). CMF-019 at 10 µM displayed no significant rescue despite trending towards significance (4.38 ± 1.48%, ns) (Figure 3).

**FIGURE 4:**
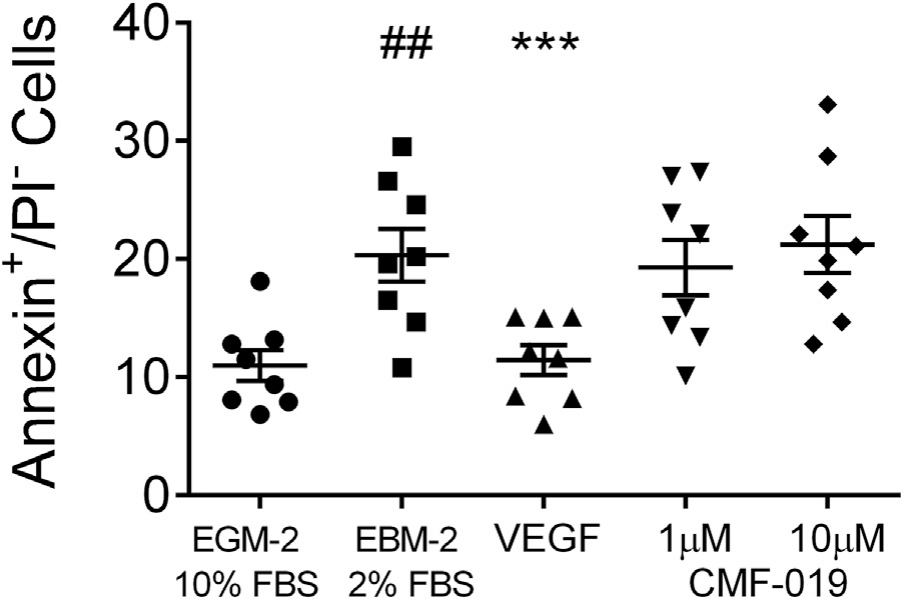
Dot plot of the percentage of annexin^+^/PI^−^ human PAECs in each experimental condition with mean ± SEM data superimposed. Cells were incubated in EGM-2 with 10% FBS alone (“growth factor control”), EGM-2 with 2% FBS alone (serum starvation) or EGM-2 with 2% FBS and 18hrs treatment with rhVEGF (10 ng/ml) or CMF-019 (1–10 μM). Growth factor and serum starvation (EBM-2 2% FBS) significantly increased the percentage of annexin^+^/PI^−^ human PAECs and this could be rescued by 10 ng/ml rhVEGF. CMF-019 did not rescue. Matched ANOVA comparing each condition to the EBM-2 2% FBS condition ***p < 0.001. ## indicates *p* < 0.01 compared to the “healthy” control (EGM-2 10% FBS).

In control experiments assessing apoptotic responses and rescue to growth factor and serum starvation (Figure 4), growth factor and serum starvation significantly increased the percentage of annexin^+^/PI^−^ cells (9.33 ± 1.94%, ^##^ *p* < 0.01) compared to the EGM-2 10% FBS “healthy” control and rhVEGF was able to completely rescue this (8.86 ± 1.07%, ***p < 0.001). However, CMF-019 at 1 µM (1.04 ± 0.86%, ns) and 10 µM (−0.91 ± 0.86%, ns) displayed no rescue in these conditions.

### CMF-019, a G Protein Biased Small Molecule, Reduced Peripheral Artery Pressure *In Vivo*

**FIGURE 5:**
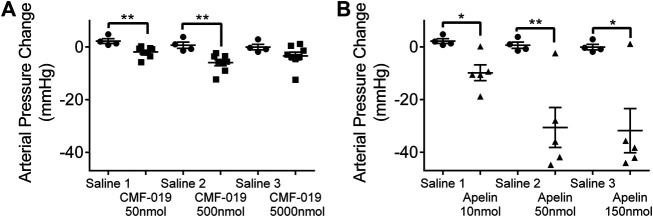
The arterial pressure change in response to CMF-019 and [Pyr^1^]apelin-13 *in vivo.* Decreases in arterial pressure in anaesthetized male Sprague-Dawley rats to **(A)** intravenous CMF-019 potassium salt (■, *n* = 8) and **(B)** [Pyr^1^]apelin-13 (apelin, ▲, *n* = 5) compared to saline (●, *n* = 4) control. Each dose was compared by a Student’s t-test to its corresponding saline control as doses were administered cumulatively (*p < 0.05, **p < 0.01).

Measured by catheterization of the femoral artery, bolus CMF-019 administration via the jugular vein revealed reproducible peripheral reduction in femoral artery pressure compared to saline at 50 nmol (4.16 ± 1.18 mmHg, **p < 0.01) and 500 nmol (6.62 ± 1.85 mmHg, **p < 0.01) (Figure 5A). At the highest dose of 5000 nmol the response was not significant. [Pyr^1^]apelin-13 produced a larger dose dependent decrease in blood pressure at all doses administered (10 nmol 12.06 ± 3.51 mmHg, *p < 0.05; 50 nmol 31.24 ± 8.70 mmHg, **p < 0.01 and 150 nmol 31.75 ± 9.53 mmHg, *p < 0.05) (Figure 5B).

### Cardiac Responses *In Vivo* to CMF-019 and [Pyr^1^]Apelin-13

**FIGURE 6:**
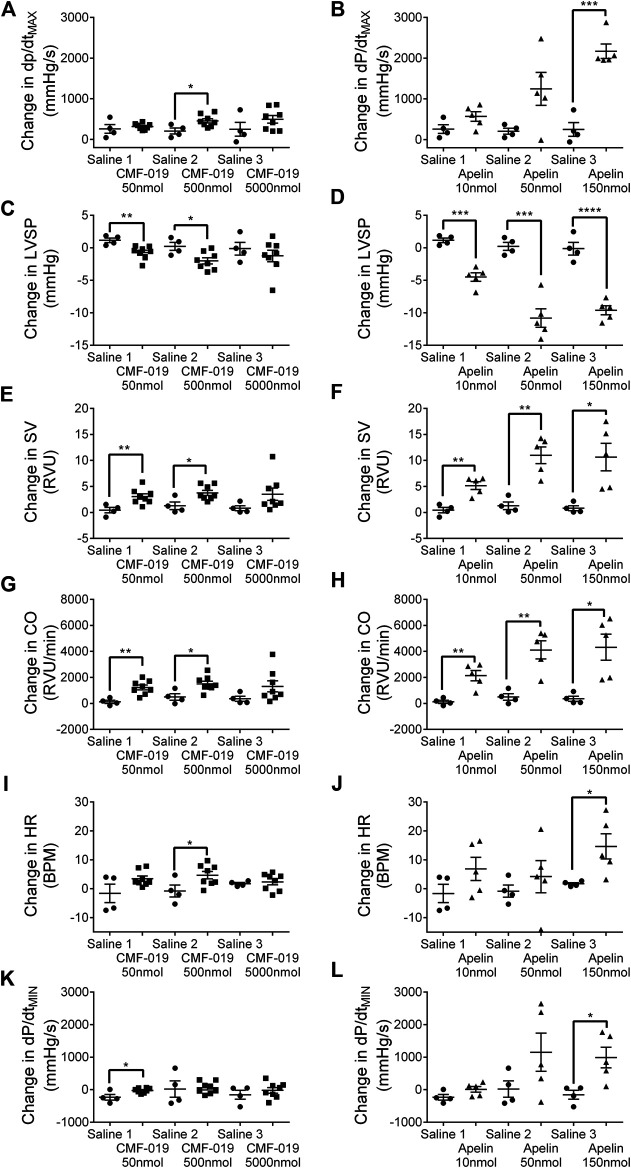
Cardiovascular responses to CMF-019 and [Pyr^1^]apelin-13 *in vivo*. Graphs showing changes in contractility (dP/dt_MAX_; **A–B**) left ventricular systolic pressure (LVSP; **C–D**), stroke volume (SV; **E–F**), cardiac output (CO; **G–H**), heart rate (HR; **I–J**) and relaxation (dP/dt_MIN_; **K-L**) for CMF-019 potassium salt (■, *n* = 8, **A and C, E, G**, **I, K**) and [Pyr^1^]apelin-13 (apelin, ▲, *n* = 5, **B, D, F, H, J, L**) compared to saline (●, *n* = 4, **A–H**) when injected intravenously into anaesthetized male Sprague-Dawley rats. Each dose was compared by a Student’s t-test to its corresponding saline control as doses were administered cumulatively (*p < 0.05, **p < 0.01, ***p < 0.001, ****p < 0.0001).

CMF-019 increased cardiac contractility (500 nmol 251 ± 89 mmHg/s, *p < 0.05), stroke volume (50 nmol 2.63 ± 0.82 RVU, **p < 0.01; 500 nmol 2.48 ± 0.87 RVU, *p < 0.05), cardiac output (50 nmol 1,097 ± 284 RVU/min, **p < 0.01; 500 nmol 1,012 ± 340 RVU/min, *p < 0.05) and produced a small elevation in heart rate (500 nmol 5.46 ± 2.32 BPM, *p < 0.05) (Figure 6). CMF-019 decreased LVSP (50 nmol 1.88 ± 0.57 mmHg, **p < 0.01; 500 nmol 2.23 ± 0.80 mmHg, *p < 0.05) in concert with the arterial pressure. A very small decrease in lusitropy was also observed (50 nmol 210 ± 70 mmHg/s, *p < 0.05). Similar to CMF-019, [Pyr^1^]apelin-13 increased cardiac contractility (150 nmol 1920 ± 178 mmHg/s, ***p < 0.001), stroke volume (10 nmol 4.72 ± 0.98RVU, **p < 0.01; 50 nmol 9.70 ± 1.93RVU, **p < 0.01; 150 nmol 9.84 ± 3.02 RVU, *p < 0.05), cardiac output (10 nmol 2,008 ± 464 RVU/min, **p < 0.01; 50 nmol 3,618 ± 818 RVU/min, **p < 0.01; 150 nmol 3,961 ± 1154 RVU/min, *p < 0.05) and decreased LVSP (10 nmol 5.66 ± 0.81 mmHg, ***p < 0.001; 50 nmol 11.02 ± 1.70 mmHg, ***p < 0.001; 150 nmol 9.47 ± 1.16 mmHg, ****p < 0.0001). [Pyr^1^]apelin-13 produced a small elevation in heart rate (150 nmol 12.94 ± 4.95 BPM, *p < 0.05) and a small decrease in lusitropy was observed (150 nmol 1,143 ± 378 mmHg/s, *p < 0.05) which was consistent with CMF-019 (Figure 6). The time course for the effect of both compounds was similar. No adverse effects were observed at any of the doses administrated.

### CMF-019 Did Not Desensitize the Apelin Receptor *In Vivo*

**FIGURE 7:**
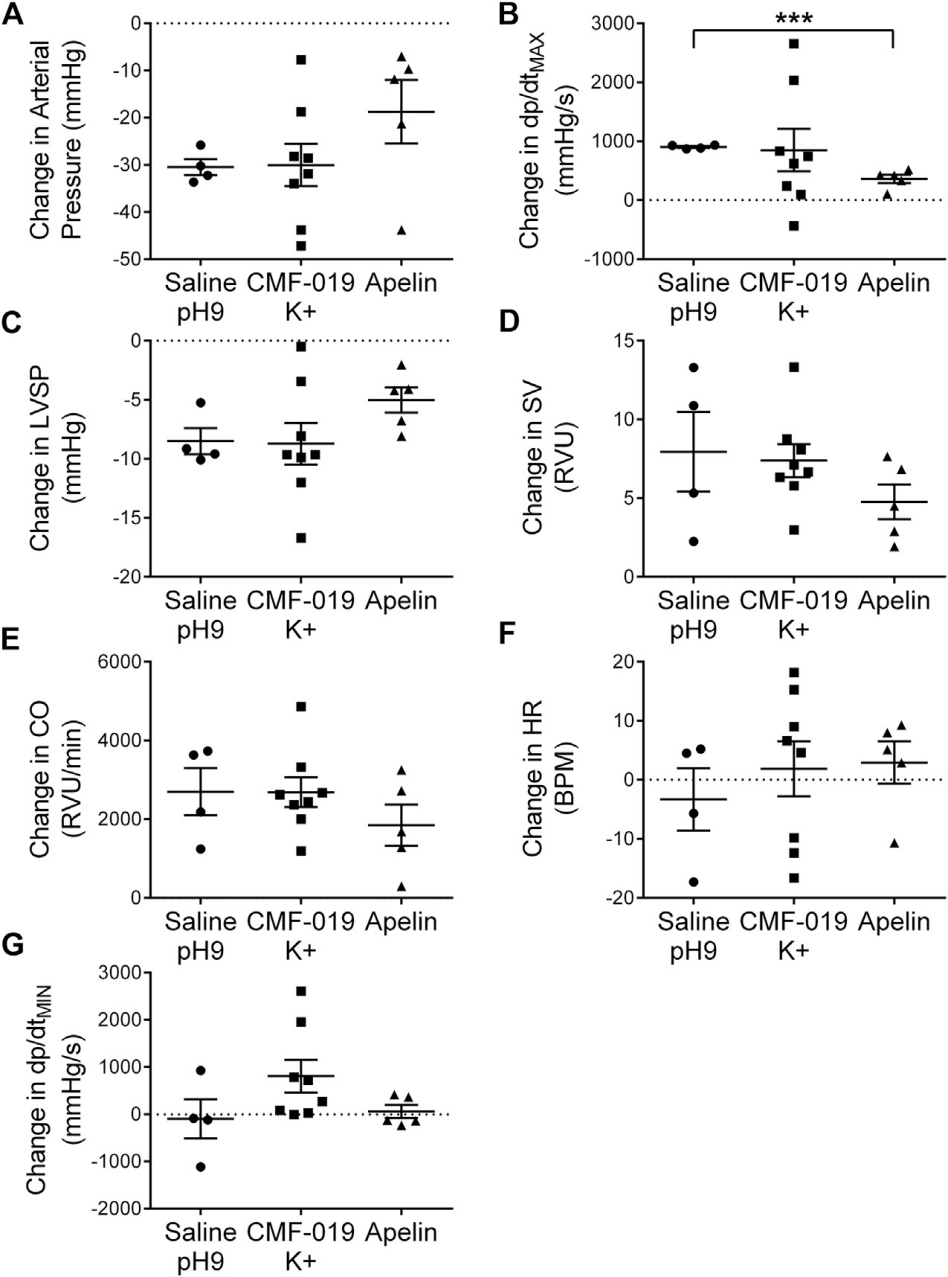
Cardiovascular responses to [Pyr^1^]apelin-13 at 50 nmol administered as a fourth dose following three successive doses of either saline pH9, CMF-019 or [Pyr^1^]apelin-13 *in vivo*. Graphs show changes in arterial pressure **(A)**, contractility (dP/dt_MAX_) **(B)**, left ventricular systolic pressure (LVSP; **C**), stroke volume (SV; **D**), cardiac output (CO; **E**), heart rate (HR; **F**) and relaxation (dP/dt_MIN_) **(G)** to 50 nmol [Pyr^1^]apelin-13 in anaesthetized male Sprague-Dawley rats previously administered with three doses of either saline (●, *n* = 4) or CMF-019 potassium salt (■, *n* = 8) or Pyr^1^]apelin-13 (▲, *n* = 5). The response to [Pyr^1^]apelin-13 after CMF-019 or [Pyr^1^]apelin-13 was compared by a Student’s t-test to that after saline control (***p < 0.001).

To study desensitization at the apelin receptor *in vivo*, a fourth dose of [Pyr^1^]apelin-13 at 50 nmol was administered to the saline, CMF-019 and [Pyr^1^]apelin-13 treatment groups (Figure 7). The *x*-axis denotes whether the preceding three doses were either saline or increasing doses of CMF-019 or [Pyr^1^]apelin-13. The responses to [Pyr^1^]apelin-13 in CMF-019 treated animals compared to saline treated animals were not significantly different for any parameter indicating that CMF-019 did not significantly desensitize the receptor compared to saline. In contrast there was a trend for the response to 50 nM [Pyr^1^]apelin-13 to be blunted in for all parameters following the three successive [Pyr^1^]apelin-13 doses that reached significance for the contractility response (*p* < 0.001) consistent with desensitization.

## Discussion

### CMF-019 Modified Disease in a Human PAEC Apoptotic Model

In the advanced stages of PAH, pathological remodeling of pulmonary vessels occurs including endothelial proliferation and the development of distinctive plexiform lesions which may in part be driven by imbalances in apelin signaling (Andersen et al., 2011; Yang et al., 2015). However, in early disease it is thought that endothelial cell apoptosis, leading to vascular dysfunction may drive onset (Wilson et al., 1992; Sakao et al., 2005; Rabinovitch, 2012). Apelin has been suggested to mitigate these effects and promotes survival of pulmonary vascular endothelial cells (Alastalo et al., 2011; Kim et al., 2013). Here we have studied the ability of the G protein biased small molecule apelin agonist, CMF-019, to promote human PAEC survival in response to apoptotic stimulation with TNFα/CHX. TNFα may prevent apoptosis of endothelial cells through activation of the NF-κB pathway, however, in conditions of global protein synthesis suppression, such as, with concurrent application of CHX, signaling through TNF-R1 dominates leading to JNK phosphorylation and induction of apoptosis (Wajant et al., 2003).

Both CMF-019 and rhVEGF rescued endothelial cells from TNFα/CHX induced apoptosis. The rescue with CMF-019 was approximately 50% of that observed for the positive control, rhVEGF, and supports a role for apelin agonists in preventing endothelial damage in healthy human PAECs when challenged with an apoptotic stimulus. The mechanism by which this occurs is not well characterized, however, both [Pyr^1^]apelin-13 and ELA-32, endogenous agonists of the apelin receptor (Read et al., 2019), promote ERK1/2 phosphorylation in human PAECs (Yang et al., 2017), while some apelin isoforms have been shown to downregulate the JNK and p-38 pathways in osteoblasts (Tang et al., 2007) and neurons (Liu et al., 2018). These pathways both have known roles in apoptosis. Furthermore, there is evidence that apelin regulates myocyte enhancer factor 2, which in turn can activate miR-424/miR-503 and genes contributing to endothelial cell homeostasis. This axis may also be beneficial in PAH by acting on pulmonary arterial smooth muscle cells. Here it inhibits the expression of fibroblast growth factor 2 and its receptor, and thus, exerts anti-proliferative effects, potentially preventing their over-proliferation and subsequent vessel muscularization (Alastalo et al., 2011; Kim et al., 2013; Helenius et al., 2015; Kim et al., 2015). Finally, agents that activate apelin expression or act as downstream effectors of apelin signaling have demonstrated beneficial effects in PAH animal models (Kim et al., 2013; Spiekerkoetter et al., 2013; Bertero et al., 2014; Nickel et al., 2015).

Interestingly, CMF-019 did not rescue apoptosis induced by serum and growth factor starvation in the control arm of the experiment, although the positive control rhVEGF did. This suggests that rescue from serum and growth factor starvation is through a different mechanism to rescue from TNFα/CHX induced apoptosis. Growth factor and serum starvation has been widely observed in various endothelial cell lines (Karsan et al., 1997; Gerber et al., 1998b; Raymond et al., 2004; Date et al., 2005; Ueda et al., 2005; Gama Sosa et al., 2016) and the mechanisms for rescue with rhVEGF are thought to occur by both upregulation of the MAPK/ERK pathway alongside downregulation of JNK pathway (Gupta et al., 1999), leading to enhanced bcl-2 and decreased bax signaling (Karsan et al., 1997; Gerber et al., 1998a; Ueda et al., 2005). In contrast, TNFα/CHX stimulates apoptosis primarily through the TNF-R1 leading to induction of the JNK and p-38 MAPK pathways. On balance there is greater evidence of apelin promoting ERK/MAPK signaling, especially in PAECs, and perhaps activation of this pathway was more efficacious in rescuing from JNK/p-38 mediated apoptosis induced by TNFα/CHX, rather than the multiple pathway mechanisms of apoptosis induced by serum and GF starvation.

This fact that CMF-019 rescued only in the TNFα/CHX condition may suggest it as a potential disease modifier in that it prevented endothelial cell apoptosis in conditions of a severe stimulus but did not have an effect in response to the weaker serum and GF starvation stimulus. Similar results were observed with the cyclic peptide, MM07, also a G protein biased apelin agonist which was effective in preventing PAH onset in a rat MCT model, suggesting a potential mechanism for modification of disease onset (Yang et al., 2019). Although [Pyr^1^]apelin-13 was administered in similar cell apoptosis experiments (data not shown), no rescue was observed and this was thought to be due either to proteolytic breakdown or high plasma protein binding in serum over the prolonged 18 h incubation time. Protease inhibitors cannot be used in this assay to prevent apelin breakdown as these would confound it by negating protein synthesis inhibition by CHX (unpublished observation).

### CMF-019 Reduced Peripheral Artery Pressure and Increased Cardiac Output *In vivo*

CMF-019 administered by bolus intravenous injection through a jugular vein cannula in normotensive male Sprague-Dawley rats induced both a reduction in pressure recorded in the femoral artery and cardiac responses, as did [Pyr^1^]apelin-13. Consistent with cardiac responses that we have previously reported to CMF-019 and [Pyr^1^]apelin-13 (Read et al., 2016), in this study both apelin agonists displayed reproducible enhancement of contractility, stroke volume and cardiac output. The maximum response to CMF-019 was smaller than that observed for [Pyr^1^]apelin-13 and this was likely due to the limited concentrations of CMF-019 that could be attained *in vivo* as previously stated. In our previous report CMF-019 had little effect on LVSP with a small increase seen at the highest dose. We postulated that the lack of effect of CMF-019 may be explained either by limited solubility or that vasodilatation resulting from apelin receptor activation may be a β-arrestin mediated response. We therefore extended the protocol in this study to also measure pressure changes in the femoral artery. In the current study we observed a small decrease in LVSP with CMF-019 consistent with the larger decreases obtained with [Pyr^1^]apelin-13. Crucially, CMF-019 induced a reduction in femoral artery pressure following administration which has not been previously demonstrated. This response most likely reflects a decrease in peripheral resistance, consistent with CMF-019, like [Pyr^1^]apelin-13, acting as vasodilators, as both molecules increase stroke volume. It has been suggested that longer apelin peptides, such as apelin-17, display β-arrestin bias by reaching deeper within the apelin binding pocket and through these contacts they both internalize the receptor and signal to produce vasodilatation (El Messari et al., 2004; Iturrioz et al., 2010; Ceraudo et al., 2014). This theory for β-arrestin bias seems likely and we observe that smaller and cyclic peptides, such as MM07, possess G protein bias, while the small molecule CMF-019 displays the greatest G protein bias we have observed (Read et al., 2016). However, from our results this does not correlate with a reduced ability to produce vasodilatation. In fact, given the marked bias of CMF-019 towards the G protein pathway, it suggests that vasodilatation is possible without engagement of β-arrestin signaling and receptor internalization. This is further supported by the sustained vasodilatation observed in humans *in vivo* with MM07 (Brame et al., 2015).

### CMF-019 Did Not Desensitize the Apelin Receptor *In Vivo*

It has previously been shown that CMF-019 displays weak activity in recruiting β-arrestin and internalizing the apelin receptor *in vitro* (Read et al., 2016). Therefore, to assess the ability of CMF-019 to internalize the apelin receptor *in vivo*, a protocol was devised whereby subsequent to the three doses of saline or three increasing doses of CMF-019 or [Pyr^1^]apelin-13 a fourth dose comprising of [Pyr^1^]apelin-13 50 nmol was administered to all saline, CMF-19 and [Pyr^1^]apelin-13 treated animals. These fourth doses were compared to assess whether there was any desensitization of the response as a consequence of the previous doses of CMF-019 or [Pyr^1^]apelin-13 administered. Although this study was limited by the number of animals that could be used and a high variance in the 50 nmol [Pyr^1^]apelin-13 responses, there is a clear trend across the parameters. There was very little difference in responses to 50 nmol [Pyr^1^]apelin-13 in any parameter when they had received saline or CMF-019 for the first three doses. In contrast the increase in contractility produced by 50 nM [Pyr^1^]apelin-13 following saline was significantly attenuated following the three doses of [Pyr^1^]apelin-13 and there was a trend for other parameters to be blunted. A limitation of this study is that the maximum response induced by CMF-019 was lower than that to [Pyr^1^]apelin-13 however overall the data suggested that CMF-019 did not desensitize the apelin receptor *in vivo*.

## Conclusion

The identification of CMF-019, the first G protein biased small molecule apelin agonist, represents an advance in the development of small molecule apelin agonists for use as experimental tool compounds for *in vitro* and *in vivo* study. This is confirmed by the fact it is already commercially available and methods to improve the synthesis of the molecule have been attempted (Trifonov et al., 2018). Furthermore, it has potential as a starting point or stimulus for the development of newer biased small molecule therapeutics at the apelin receptor with improved pharmacokinetic profiles (Narayanan et al., 2020).

In this study, we have shown that CMF-019 is able to rescue endothelial cell apoptosis that has been shown to be a driver of early PAH pathogenesis. Moreover, we have demonstrated that CMF-019 is able to induce vasodilatation despite its pronounced G protein bias, in addition to cardiac inotropy. Overall, this study supports further investigation of novel G protein biased apelin agonists such as CMF-019 but with improved pharmacokinetics as potential therapeutics in PAH.

## Data Availability

The raw data supporting the conclusions of this article will be made available by the authors, without undue reservation.
